# Leveraging the lymphohematopoietic graft-versus-host reaction (LGVHR) to achieve allograft tolerance and restore self tolerance with minimal toxicity

**DOI:** 10.1093/immadv/ltad008

**Published:** 2023-05-13

**Authors:** Megan Sykes

**Affiliations:** Columbia Center for Translational Immunology, Department of Medicine, Department of Microbiology and Immunology and Department of Surgery, Columbia University, New York, NY, USA

**Keywords:** mixed chimerism, Type I diabetes, autoimmunity, intestinal transplantation, tolerance

## Abstract

Mixed allogeneic chimerism has considerable potential to advance the achievement of immune tolerance to alloantigens for transplantation and the restoration of self-tolerance in patients with autoimmune disease. In this article, I review evidence that graft-versus-host (GVH) alloreactivity without graft-vs-host disease (GVHD), termed a lymphohematopoietic graft-vs-host reaction (LGVHR), can promote the induction of mixed chimerism with minimal toxicity. LGVHR was originally shown to occur in an animal model when non-tolerant donor lymphocytes were administered to mixed chimeras in the absence of inflammatory stimuli and was found to mediate powerful graft-vs-leukemia/lymphoma effects without GVHD. Recent large animal studies suggest a role for LGVHR in promoting durable mixed chimerism and the demonstration that LGVHR promotes chimerism in human intestinal allograft recipients has led to a pilot study aiming to achieve durable mixed chimerism.

## Achieving allograft tolerance via mixed chimerism

Achievement of immune tolerance to alloantigens and restoration of self-tolerance are major goals in transplantation and autoimmune diseases, respectively. In this article, I discuss the ability of mixed allogeneic chimerism to achieve these goals and the potential role of lymphohematopoietic graft-vs-host reactions in achieving mixed chimerism with minimal toxicity.

Responses to allogeneic major histocompatibility (MHC) antigens pose the single greatest barrier to organ allograft acceptance and promote severe graft-versus-host disease (GVHD) in hematopoietic cell transplantation (HCT). Approximately 1–3% of an individual’s T cell repertoire recognizes an allogeneic donor’s HLA [1], making it a colossal barrier that is difficult to control without paralyzing the entire immune system. Immune tolerance is therefore the ‘holy grail’ in organ transplantation, as it would avoid the many complications of long-term immunosuppressive therapy while simultaneously preventing the late graft loss that currently limits clinical transplantation.

Only two approaches have been used successfully to achieve allograft tolerance so far in humans. The first is planned weaning and withdrawal of immunosuppressive therapy, which has been more successful in liver than any other type of transplant and ultimately succeeds in only a fraction of highly selected long-term liver allograft recipients. Success rates are approximately 22% in the small subset of patients who qualify for withdrawal [2–5], but this success rate drops to 1–3% [6] when calculated on the basis of the number of patients screened rather than withdrawn. Success has been greatest in patients with a long duration of rejection-free survival on standard immunosuppression [5] and is much lower when patients are withdrawn 1–2 years after transplant [7]. Thus, this strategy is not applicable to the vast majority of liver recipients.

The second approach that has been used successfully to withdraw immunosuppression is HCT, which is the only strategy that has been used successfully in renal allograft recipients. The first trials were carried out at Massachusetts General Hospital, where we achieved allograft tolerance without GVHD using combined HLA-mismatched donor kidney and bone marrow transplantation (CKBMT) in a small group of patients [8, 9]. Trials of CKBMT at Stanford University have so far succeeded in achieving tolerance only in the less immunologically challenging HLA-identical transplant setting [10]. An approach to extensively HLA-mismatched transplantation being explored at Northwestern University has resulted in the induction of full allogeneic chimerism and is associated with a significant risk of GVHD [11]. In my view, one of the most important requirements for using hematopoietic chimerism solely for the purpose of achieving allograft tolerance and/or reversal of autoimmunity, for which many of the same requirements apply, is a minimal risk of GVHD. The reported Northwestern results do not meet this standard. A second key requirement for using HCT solely for the purpose of allograft tolerance induction is that the conditioning regimen must have minimal toxicity. While transiently high levels of immunosuppression are acceptable when permanent immunosuppression-free graft survival is achieved, the highly toxic ‘lethal’ chemotherapy/radiation-based regimens originally used for allogeneic HCT would not be justifiable in this context. Although a number of less toxic, nonmyeloablative conditioning regimens have been developed in the last few decades, these still carry significant GVHD risk [12] and none have reliably achieved durable mixed hematopoietic chimerism across extensive HLA barriers in patients or in pre-clinical non-human primate (NHP) models. These regimens still rely on GVH alloreactivity in donor hematopoietic cell grafts to eliminate residual host alloreactivity and the end result is usually full donor chimerism, with or without GVHD [12]. Thus, a major challenge needed to make HCT the standard of care in organ transplantation has yet to be met clinically or even in NHP models.

The MGH clinical trial that achieved renal allograft tolerance across HLA barriers via non-myeloablative HCT built on work we had carried out in murine models aiming to achieve graft-vs-leukemia/lymphoma effects without GVHD. We had observed that mixed chimerism could serve as a platform for donor lymphocyte infusions (DLI) that converted mixed to full donor chimerism [13] and could achieve GVL without GVHD [14] via a lymphohematopoietic GVH reaction (LGVHR) that occurs without GVHD [14–17]. The key conditions needed to achieve this outcome were: (i) initial mixed chimerism so that the recipient was tolerant of the donor and did not reject the DLI; (ii) any tissue inflammation initially induced by the conditioning had resolved before the non-tolerant DLI was given [15, 16, 18, 19]. The one-way (GVH) alloresponse under these conditions was confined to the lymphohematopoietic system [16, 17, 20–22], mediating GVL and converting mixed to full donor chimerism. Donor CD8^+^ and CD4^+^ T cells in the DLI were critical for this effect and donor CD4^+^ T cells given without CD8^+^ cells could, paradoxically, incite donor allograft rejection through an apparently cytokine-dependent mechanism that activated residual recipient T cells [23]. We showed that GVHD did not occur in this setting because tissue inflammation is needed to promote T cell migration to epithelial GVHD target tissues and this condition did not exist when a sufficient delay ­after initial conditioning was allowed before DLI administration [16]. We subsequently attempted to apply these principles in clinical trials in both the HLA-identical [24–27] and HLA-mismatched [28–30] settings in patients with hematological malignancies. While many of the patients had bulky, advanced malignancies and were not cured by this approach, several patients had remarkable outcomes, including one who developed durable mixed chimerism across HLA barriers [28] and others who converted from mixed to full donor chimerism following DLI [30]. Our murine data demonstrated the importance of recipient APCs and particularly their expression of MHC class I and class II in mixed chimeras for the priming of the LGVHR and the achievement of GVL, even for tumors that lacked class II MHC expression [15, 20, 22]. This class II requirement reflected a need for CD4 help in order for CD8 effector cells to differentiate and induce effective LGVHR-dependent GVL effects [20]. This requirement did not exist in the GVHD-conducive inflammatory environment of freshly irradiated recipients, in which GVH-reactive CD8 CTLs differentiated in a CD4-independent fashion and also mediated GVHD in addition to GVL [20, 21]. Results in these clinical trials were similarly consistent with an optimal GVL effect in the presence of mixed rather than full donor chimerism [24]. Importantly, these studies provided safety data and proof of principle that transient or permanent mixed ­chimerism could be achieved across HLA barriers without GVHD [28, 30].

One of the first regimens tested in the HLA-mismatched setting using the above approach resulted in only transient chimerism without GVHD. NHP studies had meanwhile shown that transient MHC-mismatched mixed chimerism could be sufficient to achieve renal allograft tolerance in a large fraction of animals [31]. Furthermore, we found that transient chimerism could permit HLA-identical donor renal allograft tolerance in multiple myeloma patients receiving a related regimen [32–34]. With this information in hand, the transient chimerism regimen used in HLA-mismatched patients with malignancies thereby provided the missing safety data that justified the first trial of mixed chimerism for tolerance to HLA-mismatched kidneys in patients without any malignant disease. While renal allograft tolerance was achieved for years in 7 of 10 subjects [9], chimerism was only transient in these patients. Mechanisms of tolerance included gradual peripheral deletion of donor-reactive T cells [35, 36] and an early role for expanded donor-specific Tregs [37].

## Restoring self tolerance via hematopoietic chimerism

Animal studies have demonstrated that mixed chimerism also has the potential to reverse autoimmune diseases [38–40]. Treatment of Type I diabetes (T1D) currently requires life-long insulin therapy and is associated with significant morbidities. Since recipient beta cells have been largely destroyed in T1D patients, replacement of patient beta cells is an important component of any curative therapy. Durable mixed chimerism has been shown to achieve tolerance to donor islet allografts [41–46], avoiding the need for long-term immunosuppressive therapy that currently limits the application of clinical islet transplantation to the small subset of T1D patients with life-threatening complications such as hypoglycemic unawareness. Furthermore, in the NOD mouse model of autoimmunity, durable but NOT transient mixed chimerism has been shown to reverse the autoimmune process [46–48]. Our studies demonstrated that durable mixed chimerism achieved with non-myeloablative conditioning resulted in tolerance to donor islet allografts while simultaneously allowing syngeneic islet graft acceptance without infiltrates in NOD mice with advanced autoimmune diabetes [46]. This powerful demonstration of reversal of autoimmunity was found to be mediated by regulatory cells of the non-diabetes-prone allogeneic donor in the mixed chimeras (J. Spinelli, J. Kurtz, and M. Sykes, unpublished data). In human immune systems generated from bone marrow CD34+ cells of type I diabetic (T1D) donors, we have demonstrated a defect in Treg differentiation of an islet autoreactive TCR (R. Madley and M. Sykes, unpublished data), suggesting that durable mixed chimerism may have the potential to reverse autoimmunity by a similar dominant Treg-mediated mechanism in human T1D patients.

Based on the potential of durable mixed chimerism to simultaneously reverse autoimmunity and allow donor islet replacement without the need for chronic immunosuppression, we have developed an NHP model aiming to achieve durable mixed chimerism with minimal conditioning. We have achieved this in a cohort of animals (D. Eisenson, M. Sykes, K. Yamada et al, unpublished data) and demonstrated tolerance to donor kidney allografts in an MHC haplotype-mismatched setting. Although mechanistic studies have not yet been carried out, it seems likely that the large number of GVH-alloreactive donor T cells carried in the PBSC graft plays a significant role in the achievement of durable chimerism and tolerance in this model. This regimen is based on a similar one, involving only low dose (100 cGy) TBI, partial peripheral T cell depletion, and a short course of calcineurin inhibitor, that achieved low levels of durable mixed chimerism across MHC barriers in a porcine model. Unlike the monkey model, in which high levels of chimerism persisted in both the myeloid and lymphoid cell lineages, the myeloid chimerism in the porcine model was minimal, while the durable chimerism was seen mainly in the T cell lineage [49–53]. GVH-reactive T cells persisted in these animals and were suppressed by a recipient cell population [54].

Recently, our group has demonstrated that multilineage mixed chimerism develops spontaneously in patients receiving intestinal allografts, and particularly in those receiving multivisceral transplants that include liver, stomach, and pancreas in addition to the intestine. ‘Macrochimerism’, which we defined as a peak level of T cell chimerism >4%, is associated with reduced rates of graft rejection and *de novo* donor-specific antibody production compared to patients lacking macrochimerism [55–57]. The chimerism in these patients reflects engraftment of hematopoietic stem and progenitor cells (HSPCs) that we found to be carried within the intestinal graft mucosa, liver, and associated lymphoid tissue [56]. Consequently, donor HSPCs are detected in the recipients’ bone marrow and blood chimerism involves multiple lineages, including myeloid and lymphoid cell types. Both B cells and T cells of donor origin include prominent naïve and ‘recent thymic emigrant’ components [56], respectively, suggesting that they develop *de novo* in the recipient after engrafting in the recipient’s bone marrow. Using a high throughput TCRβ CDR3 sequencing approach that we have developed to identify and track alloresponses in human transplant recipients, we were able to demonstrate that donor T cells pre-existing in the intestinal allograft mucosa and mediating an LGVHR appear to play a major role in allowing these beneficial outcomes [57]. We have found that recipient APCs rapidly enter the intestinal allograft mucosa and that this entry is associated with early expansion of GVH-alloreactive donor T cells within the allograft [55]. These GVH-reactive donor T cells are presumably microbe-reactive tissue-resident memory cells that have cross-reactive recognition of recipient alloantigens. In association with this activation and expansion in situ that results from the entry of recipient APCs into the mucosa, GVH-reactive T cells leave the allograft and enter the peripheral circulation, where their levels peak at one to two weeks following transplantation [57]. In most cases in our series, GVHD was absent, classifying these events as LGVHR. When we have examined recipient bone marrow at later time points, donor T cells have been detected along with donor hematopoietic cells of other lineages, including HPSCs. Single-cell RNA sequencing combined with TCR sequencing allowed us to demonstrate that the donor T cells detected in recipient marrow include GVH-reactive clones with cytotoxic effector function [57]. This direct demonstration of LGVHR in the circulation and bone marrow of intestinal transplant recipients provides proof of principle of the potential of LGVHR to promote mixed chimerism in humans. Based on these results, we have initiated a trial of infusion of additional donor CD34+ cells harvested from the bone marrow at the time of peak LGVHR in the circulation. Our goal is to exploit the LGVHR to achieve higher levels of more permanent donor HSPC engraftment and consequently durable mixed chimerism, with the potential to promote donor-specific tolerance.

The demonstrated ability of LGVHR to promote durable mixed chimerism in humans suggests an additional potential benefit, namely the destruction of pre-existing autoreactive recipient T cells in patients with autoimmune disease. As summarized above, LGVHR destroys recipient hematopoietic cells and we suspect that the association between reduced allograft rejection and LGVHR in intestinal transplant recipients reflect that ability of GVH-reactive T cells to destroy or suppress donor-reactive (HVG) recipient T cells. Likewise, pre-existing autoreactive T cells might be destroyed or attenuated by an LGVHR, particularly one that converts mixed into full donor chimerism, as demonstrated in the murine model. Although this mechanism would not be expected to exhibit specificity for autoreactive T cells but instead to globally destroy recipient hematopoietic cells, it could achieve a functioning donor immune system without GVHD.

**Figure 1:**
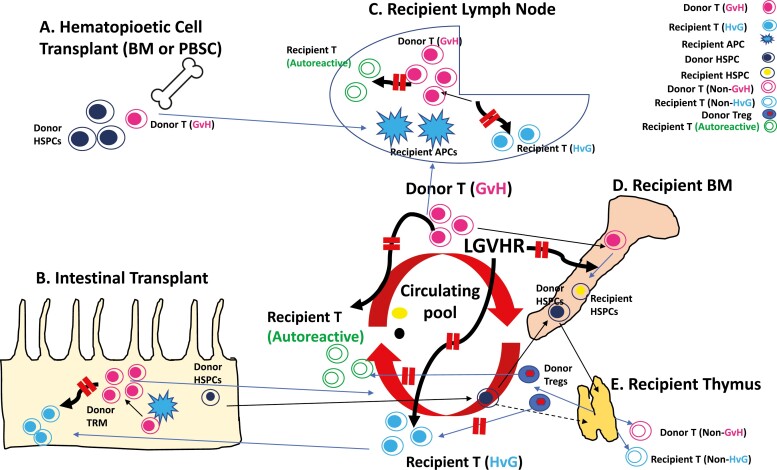
Mechanisms by which lymphohematopioetic GVHR (LGVHR) may promote tolerance to allografts and reverse autoimmunity. LGVHR have been demonstrated following: A) Hematopoietic cell transplantation (HCT) [13–16, 59–61], in which GVH-reactive T cells are included in a bone marrow or PBSC transplant product that contains donor hematopoietic stem and progenitor cells (HSPCs). GVH-reactive T cells enter the circulation and the recipient lymph nodes, where they expand in response to recipient APCs. Donor HSPCs enter the circulation and the recipient bone marrow; and B) Intestinal transplantation, in which the intestinal mucosa and associated structures (as well as the donor liver, when co-transplanted with intestine) contain HSPCs [56] and tissue-resident memory T cells (TRMs), some of which are GVH-crossreactive. Recipient APCs enter the graft mucosa early post-transplant, where they induce expansion and migration into the circulation of GVH-crossreactive TRMs [55, 57]. Donor HSPCs carried in the graft also enter the circulation and enter the recipient’s bone marrow [57]. C) Depicts expansion in response to recipient APCs in recipient lymph node of GVH-reactive T cells that exited from the intestinal allograft or were carried in an HCT. From there, expanded GVH-reactive T cells re-enter the circulation and other lymphoid tissues and migrate to recipient bone marrow. D) Depicts events in the recipient bone marrow following entry of donor-derived GVH-reactive T cells and HSPCs from either an HCT or an intestinal transplant. GVH-reactive effector T cells entering the recipient bone marrow express cytotoxic and cytokine effector molecules (such as perforin, granzyme A, TNF and Th1 and Th17 transcription factors) and attack recipient HSPCs to create ‘space’ for donor HSPC engraftment [57]. They may also attack and kill recipient lymphocytes in the bone marrow and elsewhere, thereby eliminating HvG-reactive T cells and autoreactive T cells. Donor HPSCs in the bone marrow co-exist with recipient HPSCs to produce mixed hematopoietic chimerism in the circulation and send progeny to the recipient thymus (E), generating recent thymic emigrants of both donor and recipient origin [56] that are mutually tolerant of one another. The thymus may also generate donor Tregs that are more effective than recipient Tregs in suppressing autoreactive T cells [46](J. Spinelli, J. Kurtz and M. Sykes, unpublished data), thereby reversing autoimmune disease.

In summary (Fig. 1), LGVHR, first demonstrated in the mouse model and more recently shown to be operative in humans, has clearly demonstrated the ability to promote donor chimerism and to mediate GVL without GVHD. LGVHR has the additional potential to correct autoimmune disease via two mechanisms: (i) by promoting induction of mixed hematopoietic chimerism that leads to the generation of Tregs that suppress autoreactive recipient T cells; (ii) by destroying autoreactive recipient lymphocytes [58]. The proofs of principle provided by achievement of durable mixed chimerism without GVHD in NHPs and humans suggest that this approach deserves further exploration in animal models and, ultimately, in patients with autoimmune disease.

## Abbreviations:

APC: Antigen-presenting cell
CDR3: Complementarity-determining region
HPSC: Hematopoietic progenitor and stem cell
HVG: Host-versus-graft
CKBMT: Combined kidney and bone marrow transplantation
DLI: Donor lymphocyte infusion
GVH: Graft-vs-host
GVHD: Graft-vs-host disease
GVL: Graft-vs-leukemia
LGVHR: Lymphohematopoietic graft-vs-host reaction
HCT: Hematopoietic cell transplantation
HLA: Human leukocyte antigen
MHC: Major histocompatibility complex
NHP: Non-human primate
NOD: Non-obese diabetic (mouse)
T1D: Type 1 diabetes
TBI: Total body irradiation
TCR: T cell receptor
TRM: Tissue resident memory
